# Prevalence and Significance of Non-conventional Antiphospholipid Antibodies in Patients With Clinical APS Criteria

**DOI:** 10.3389/fimmu.2018.02971

**Published:** 2018-12-14

**Authors:** Elena Litvinova, Luc Darnige, Amos Kirilovsky, YANN Burnel, Gonzalo de Luna, Marie-Agnes Dragon-Durey

**Affiliations:** ^1^Laboratory of Immunology, HEGP, APHP, Paris, France; ^2^Department of Biological Haematology, HEGP, APHP, Paris, France; ^3^Inserm UMR-S1140, Paris, France; ^4^Paris Descartes University, Paris, France; ^5^Immunomonitoring Plateform, HEGP, APHP, Paris, France; ^6^Internal Medicine Department, HEGP, APHP, Paris, France

**Keywords:** antiphospholipid syndrome, autoantibodies, anti-phosphatidylserine-prothrombin antibodies, lupus anticoagulant, thrombosis

## Abstract

**Background:** The biological diagnostics of antiphospholipid syndrome (APS) takes into account the persistent positivity for anticardiolipin and/or anti-β2GP1 antibodies and/or presence of lupus anticoagulant (LA). However, some non-conventional antiphospholipid antibodies have emerged that could help in the diagnosis of APS.

**Objectives:** To study the potential usefulness of non-conventional antiphospholipid antibodies in clinical practice.

**Methods:** Eighty-seven patients, aged from 15 to 92 years were included and classified in following groups: 41 patients positive for the conventional antibodies with clinical criterion of APS (31 with primary APS and 10 secondary), 17 seronegative APS (SNAPS) patients (i.e., persistent negativity for the conventional antibodies with a strong clinical suspicion of APS), 11 asymptomatic antiphospholipid antibodies carriers (i.e., persistent positivity for the conventional antibodies without clinical evidence of APS), and 18 patients presenting with a first thrombotic or obstetrical event. IgG and IgM were detected to the following antigens: phosphatidylserine/prothrombin (PS/PT) by ELISA, and phosphatidic acid, phosphatidyl-ethanolamine, phosphatidyl-glycerol, phosphatidyl-inositol, phosphatidylserine, annexin V, prothrombin by immunodot. Anti-β2GP1 IgA, and anti-β2GP1 domain 1 IgG were detected by chemiluminescence.

**Results:** Positivity for the non-conventional antibodies was correlated with APS severity; patients with catastrophic APS (CAPS) being positive for 10.7 (Median, Range: 5–14) non-conventional antibodies. 9/17 seronegative patients were positive for at least one of non-conventional antibodies. A study of non-supervised hierarchical clustering of all markers revealed that anti-PS/PT antibodies showed high correlation with the presence of LA. All patients with APS triple positivity (highest risk profile) exhibited also persistent positivity for anti-PS/PT antibodies.

**Conclusions:** Our data obtained from a prospective cohort constituted mainly by patients with primary APS, suggest that non-conventional APS antibodies may be useful for patients classified as SNAPS. They demonstrate the potential value of aPS/PT antibodies as a strong marker of APS. We propose that anti-PS/PT antibodies could be a surrogate APS biological marker of LA to classify in high-risk profile patients treated by direct oral anticoagulants (DOACs), in whom LA detection cannot be achieved.

## Introduction

The antiphospholipid syndrome (APS) is an autoimmune disorder characterized by thrombotic and/or obstetrical manifestations associated with the persistent positivity for at least one of three markers: lupus anticoagulant (LA), anti-cardiolipin antibodies (aCL) or anti-beta2-glycoprotein I (aβ2GP1) antibodies of either IgG or IgM isotype (1, 2).

The prevalence of APS is estimated at around 40–50 cases per 100,000 persons (3). APS can be primary, i.e., without any other definable disease, or secondary, i.e., associated with other diseases, the most often being systemic lupus erythematosus (4). According to the site of thrombosis and the number and size of vessels involved, APS has different kinds of clinical manifestations such as peripheral thrombosis, neurological, pulmonary, cardiac or obstetric manifestations (recurrent fetal losses or *intra utero* growth retardation) (5). The most severe form of APS, called catastrophic APS (CAPS) is characterized by multiorgan failure due to diffuse thrombotic microvasculopathy (6, 7), and counts for < 1% of APS (5).

Secondary prevention of thrombosis is based on a long-term anticoagulation therapy (8). In some severe cases, rituximab may be indicated (9). The treatment of CAPS requires an aggressive therapy using anticoagulation associated with high-dose steroids, plasma exchange or intravenous immunoglobulin (10).

Considering its high rate of thrombotic recurrence that may be efficiently prevented by prophylactic anticoagulant therapy, an accurate identification of patients with APS is crucial. Also, treatment of APS women during pregnancy leads to a very significant improvement of fetal and maternal outcomes (11, 12).

Despite progresses in the treatment, patients with APS still develop significant morbidity and mortality (13, 14), and rapid diagnosis and treatment as well as the determination of reliable prognostic markers are of ultimate importance.

Antiphospholipid antibodies (APA) are constituted by a heterogeneous group of autoantibodies directed against anionic phospholipids, phospholipid-binding plasma proteins or protein-phospholipid complexes (15). Apart from conventional biological markers (LA, aCL and aβ2GP1), numerous other markers of APS have been studied such as antibodies against phosphatidyl-ethanolamine (16), phosphatidylserine-prothrombin (17), that could be helpful in the diagnosis of so-called seronegative APS (SNAPS) (18, 19), which defines a group of patients with clinical manifestations of APS but with persistently negative aCL, aβ2GP1 antibodies and LA. Furthermore, the study of the autoantibodies' epitope specificities revealed that the β2GP1 domain I is the most specific target of APS (20). In addition, antibodies of IgA isotype, mainly against β2GP1, have been suggested as a new marker in SNAPS patients (21, 22).

In recent years, direct oral anticoagulants (DOACs) therapies directly inhibiting thrombin or factor Xa are used more frequently and many studies have shown that these drugs may influence LA testing. DOACs induce false positive results of LA detection even when their concentration is very low. Therefore, LA testing should not be performed during treatment with DOACs (23). LA is the conventional biological marker associated with the strongest risk for thrombosis and other clinical APS manifestations. Moreover, it has been demonstrated that triple positivity for conventional markers in APS patients or in asymptomatic APA carriers is associated with significantly higher risk of thrombosis than single or double positivity (24, 25). LA testing in patients treated by DOACS would necessitate discontinuing the treatment for at least 3 days but it may be unsafe. Interestingly, presence of anti-phosphatidylserine/prothrombin antibodies has been shown to be closely associated with presence of LA (26).

In this study, we aimed to explore the usefulness of several non-conventional APS markers for the diagnosis of patients with clinical manifestations of APS and SNAPS. We also wondered if anti-phosphatidylserine-prothrombin antibodies could replace the use of LA testing in patients treated with DOACs. Presence and persistence of 10 non-conventional APS markers have been studied prospectively in patients presenting with APS or thrombosis and in healthy donors.

## Materials and Methods

### Patients

Eighty seven patients were included prospectively between September 2015 and May 2017. Patients were aged from 15 to 92 years old and comprised of 50 women and 37 men (Table 1). Additionally, a group of 30 healthy donors (HD) was used as controls. The study was done in accordance with the Declaration of Helsinki and Good Clinical Practice guidelines. No additional sample from the patients was collected for the study. All patients were seen in the context of their routine care, clinically evaluated and informed about the study and about the computerization of personal health data by the same physician (LD) during the follow up of the study. The patients gave oral informed consent in accordance with French legal standards for observational studies. This study has been approved by the Institutional Review Board according to standards currently applied in France (*Commission Nationale de l'Informatique et des Libertés*”, CNIL N°1922081 from 02/02/2016).

**Table 1:** Patients' characteristics.

	**Frequency (number)**
**AGE (YEARS)**	
15–30 31–60 61–92	15% (13) 66% (57) 19% (17)
**SEX**	
Females Males	57% (50) 43% (37)
**DISEASE GROUP:**	
1. APS	47% (41)
*According to clinical signs*:	
Thrombosis: CAPS Arterial Venous Both Obstetrical morbidity only	88% (36) (3) (10) (21) (2) 12% (5)
*According to number of conventional biological markers:*	
With 1 biomarker With 2 biomarkers With 3 biomarkers	32% (13) 24% (10) 44% (18)
*Primary or secondary*:	
Primary Associated with lupus Associated with Autoimmune Hepatitis	76% (31) 22% (9) 2% (1)
2. SNAPS Thrombosis: Arterial Venous Both Obstetrical complications 3. Asymptomatic APA carriers 4. First Thrombotic/obstetrical event: Arterial Venous Both Obstetrical	20% (17) 56% (9) (1) (7) (1) 44% (8) 13% (11) 22% (18) (6) (10) (1) (1)

*APS, Anti-phospholipid syndrome; CAPS, Catastrophic anti-phospholipid syndrome; SNAPS, Seronegative anti-phospholipid syndrome; APA, Anti-phospholipid antibodies*.

Blood samples were collected and sera were analyzed. According to the clinical data and to the biological parameters, the patients were classified into the following groups: (1) APS; (2) SNAPS, i.e., patients with strong clinical suspicion of APS but persistently negative for conventional biological markers; (3) asymptomatic antiphospholipid antibodies (APA) carriers, i.e., clinically asymptomatic individuals presenting with persistent antiphospholipid antibodies positivity (discovered fortuitously, mostly during preoperative assessment of hemostasis); (4) first thrombotic event and/or obstetrical morbidity group, i.e., patients presenting with a first thrombotic or obstetrical event.

Patients were classified according to the type of clinical feature: arterial thrombosis, venous thrombosis, both, obstetrical complications or CAPS (Table 1).

Our APS cohort is composed by a majority (76%, *n* = 31) of patients presenting with primary APS whereas 24% had an APS secondary to lupus (9 patients) or other autoimmune disease [autoimmune hepatitis: 1 patient] (Table 1).

### Methods

The two different coagulation tests used to detect lupus anticoagulant (LA) according to ISTH recommendations (27) were: dilute Russell venom viper time (dRVVT) using LA1 reagents (Siemens, Germany) and aPTT using Automated APTT (Trinity Biotech, Ireland).The phospholipid dependence was confirmed by positive phospholipid-neutralizing assays for both tests with a dRVVT screen (LA1 reagent)/confirm (LA2 reagent, Siemens, Germany) ratio and a ratio of aPTT with silica (LA sensitive reagent)/Kaolin PTT (LA insensitive reagent, CK Prest, Stago, France).

Anti-CL and aβ2GP1 antibodies of IgG isotype were detected in the serum by the routinely used ELISA methods: Cardiolisa, (Theradiag, Croissy-Beaubourg, France) and QUANTA Lite® β_2_ GP1 IgG (Inova Diagnostics Werfen, Les Lilas, France). Anti-CL and aβ2GP1 antibodies of IgM isotype were detected by the immunodot technique (Cf below).

Anti-phosphatidylserine-prothrombin (aPS/PT) antibodies of IgM and IgG isotypes were measured in the serum by ELISA (Quanta Lite, INOVA Diagnostics, Werfen, Les Lilas, France).

The immunodot technique (Anti-Phospholipid 10 Dot, Generic Assays, Eurobio, Les Ulis, France) was performed in the serum using the BlueDiver Instrument (D-Tek, Ingen, Chilly Mazarin, France) allowing the detection of IgG and IgM antibodies directed against the following antigens: CL, β2GP1, phosphatidic acid (PA), phosphatidyl-ethanolamine (PE), phosphatidyl-glycerol (PG), phosphatidyl-inositol (PI), phosphatidylserine (PS), annexin V (A5), and prothrombin (PT).

Anti-β2GP1of IgA isotype and aβ2GP1 domain I antibodies were measured in the serum by BIO-FLASH Chemiluminescent Immuno Assay technology (QUANTA Flash® β_2_ GP1 IgA Inova Diagnostics Werfen, Les Lilas, France).

Prism and Medcalc Softwares were used for the statistical analysis and Genesis software (version 1.8.1) was used to perform the hierarchical clustering of APS biomarkers. The titers of APL antibodies were compared using Mann-Whitney-Wilcoxon test. Correlation between the presences of different antibodies was measured by Kendall correlation using the software R version 3.4.4 [R (28)] and the package ggcorrplot version 0.1.1 (29).

## Results

### Results of Classical APS Markers

The distribution and prevalence of the classical APS markers in our cohort are depicted in the Supplementary Figure 1.

Presence of aCL antibodies was detected in 95% patients with APS and in 45% in the group of asymptomatic APA carriers but was not detectable in the group of SNAPS and thrombosis/obstetric (Supplementary Table 1). Their levels were significantly higher in the APS patients with three conventional biomarkers than in the other patients' groups (Supplementary Figure 1A).

Presence of aβ2GP1 was detected in 51% of patients with APS and in 18% of patients from the group of asymptomatic APA carriers and was not detectable in the group of SNAPS and thrombosis/obstetric. IgG aβ2GP1 positivity was observed almost only in the group of APS patients with 3 biomarkers (Supplementary Figure 1B). The patients presenting with CAPS exhibited the highest titers of aCL and aβ2GP1 antibodies (Supplementary Figures 1C,D).

LA positivity was observed in 68% of APS patients and in 100% of patients from the group of asymptomatic APA carriers and was not detectable in the group of SNAPS and thrombosis/obstetric. Its prevalence in different groups of patients is shown in the Supplementary Table 1.

### Results of aPS/PT Antibodies

Anti-PS/PT antibodies of IgG and IgM isotypes were measured in all groups of patients and controls.

**Figure 1:**
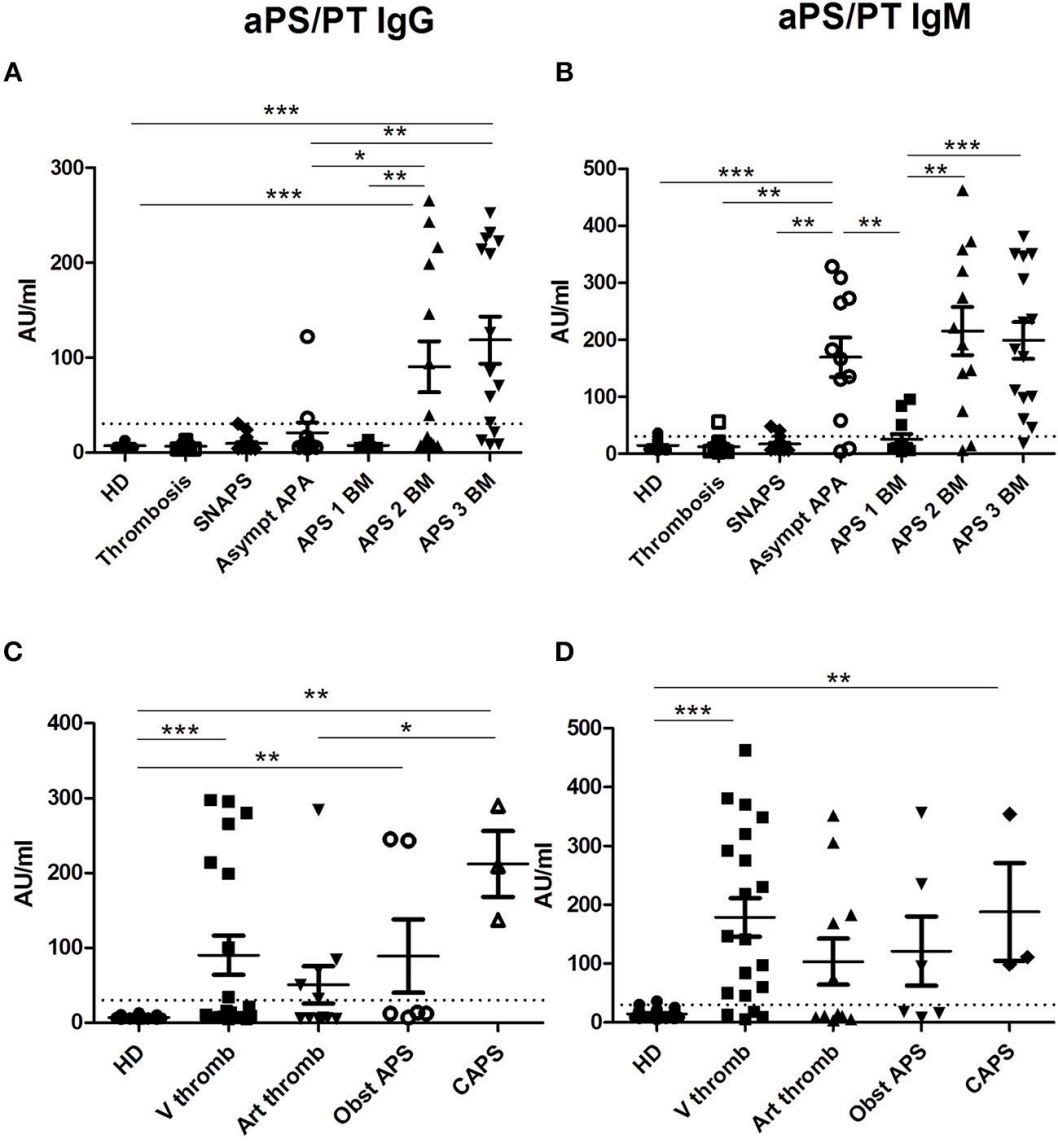
Results of anti-PS/PT antibodies. The distribution of anti-PS/PT IgG or IgM antibodies is shown in all groups of patients and in HD (**A,B** according to the disease group; **C,D** according to the clinical type of APS). The positive thresholds (30 AU/mlAU/ml) are shown by dotted lines. **(A,C)** aPS/PT antibodies of IgG isotype, **(B, D)** aPS/PT antibodies of IgM isotype. The data are presented as mean ± s.e.m. with all individual dots shown. The *p*-values were calculated using Mann-Whitney test. ^*^p < 0.05; ^**^p < 0.01; ^***^p < 0.001. *HD*, Healthy Donors*; SNAPS*, seronegative anti-phospholipid syndrome*; Asympt APA*, asymptomatic carriers of antiphospholipid antibodies; *APS 1 BM*, APS with 1 biomarker; *APS 2 BM*, APS with 2 biomarkers; *APS 3 BM*, APS with 3 biomarkers; *V thromb*, venous thrombosis; *Art thromb*, arterial thrombosis; *Obst APS*, obstetrical APS; *CAPS*, Catastrophic anti-phospholipid syndrome.

Presence of IgG aPS/PT was detected in 43.9% of patients in the group of APS, 5.6% in the group of SNAPS, 18.2% in the group of asymptomatic APA carriers. It was not detectable in the group of thrombosis/obstetric and in HD. Their levels were significantly higher in the APS patients with two and three biomarkers than in the other groups of patients (Figure 1A).

Presence of IgM aPS/PT was detected 65.8% in the group of APS, 16.7% in the group of SNAPS, 81.8% in the group of asymptomatic APA carriers, 5.3% in the group of thrombosis/ obstetric, and 6.7% in HD. Their levels were significantly higher in the groups of APS with two and three biomarkers but also in the group of asymptomatic APA carriers as compared to the other groups (Figure 1B).

The comparison of the levels of aPS/PT antibodies in APS patients according to the different types of thrombosis, showed significant differences between HD and APS patients with venous thrombosis, obstetrical morbidity and CAPS for IgG isotype (Figure 1C). For IgM isotype significant differences were observed between HD and APS patients with venous thrombosis and CAPS (Figure 1D). Patients with CAPS had statistically significant higher levels of IgG aPS/PT antibodies than patients with arterial thrombosis (*p* = 0.029, Figure 1C).

### Results of aβ2GP1 Antibodies of IgA Isotype and aβ2GP I Domain I Antibodies of IgG Isotype

**Figure 2:**
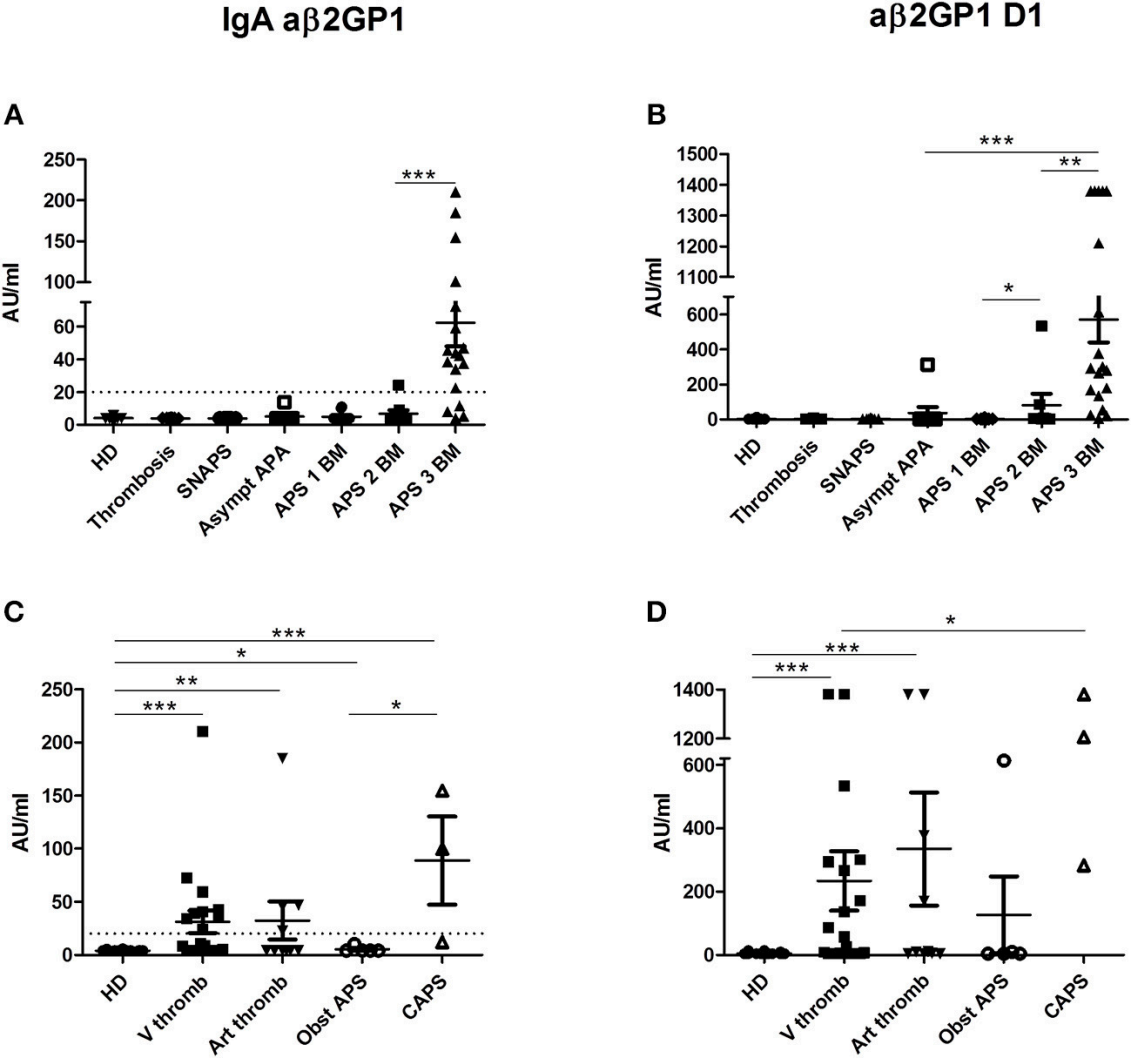
Results of anti-β2GP1 IgA and anti- β2GP1 D1 IgG antibodies. The distribution of IgA aβ2GP1 and aβ2GP1 D1 antibodies is shown in all groups of patients and in HD (**A,B** according to the disease group; **C,D** according to the clinical type of APS). The data are presented as mean ± s.e.m. with all individual dots shown. The *p*-values were calculated using Mann–Whitney test. ^*^p < 0.05; ^**^p < 0.01; ^***^p < 0.001. The limit of positivity for IgA aβ2GP1 (20 AU/ml) is shown by the dotted line **(A,C)**. The limit of positivity for aβ2GP1 D1 is 20 lAU/ml (not shown) **(B,D)**. **(A)** Statistical differences are significant between APS 3 BM and all other groups (*p* < 0.001). **(B)** In addition to differences shown in the graph, statistical differences are significant between APS 3 BM, and APS 2 BM (*p* < 0.01) and between APS 3 BM and all other groups (*p* < 0.001). *HD*, Healthy Donors*; SNAPS*, seronegative anti-phospholipid syndrome*; Asympt APA*, asymptomatic carriers of antiphospholipid antibodies; *APS 1 BM*, APS with 1 biomarker; *APS 2 BM*, APS with 2 biomarkers; *APS 3 BM*, APS with 3 biomarkers; *V thromb*, venous thrombosis; *Art thromb*, arterial thrombosis; *Obst APS*, obstetrical APS; *CAPS*, Catastrophic anti-phospholipid syndrome.

IgA aβ2GP1 were found only in patients with APS with two (31.6%) or three biomarkers (60.9%) and were negative in the other groups (Figure 2A). The levels of IgA aβ2GP1 were significantly higher in the group of APS patients with three biomarkers than those with two biomarkers (*p* = 0.0002).

IgG aβ2GP1 domain I (aβ2GP1 D1) were detected in 50% in the group of APS and in 11.1% in the group of asymptomatic APA carriers but were not detectable in the groups of SNAPS, thrombosis/obstetric and in HD (Figure 2B). Their levels were higher in the APS patients with triple positivity than those observed in patients with two biomarkers (mean = 571 vs. mean = 82 AU/ml, *p* = 0.0027). Of note, one patient from the asymptomatic APA carriers' group was highly positive for aβ2GP1 D1 (314 AU/ml, normal values < 20 AU/ml).

No correlation was found between the clinical type of APS and level of these antibodies but in CAPS patients the level of IgA aβ2GP1 was significantly higher than in patients with obstetrical APS (Figure 2C) and the level of aD1 β2GP1 in CAPS was significantly higher than in patients with venous thrombosis (Figure 2D).

### Frequency and Distribution of Other Non-conventional APS Antibodies Tested by Immunodot

Presence of other non-conventional markers of APS was searched in serum from all groups of patients and from healthy donors by a semi-quantitative analysis (immunodot).

A higher frequency of positivity or titer in patients compared to HD was observed for IgG directed against PG, PA, PI, PS, and for IgM anti-PI, PS, PA, and PT (Supplementary Figure 2). No positivity was observed for anti-PE IgG and IgM.

**Figure 3:**
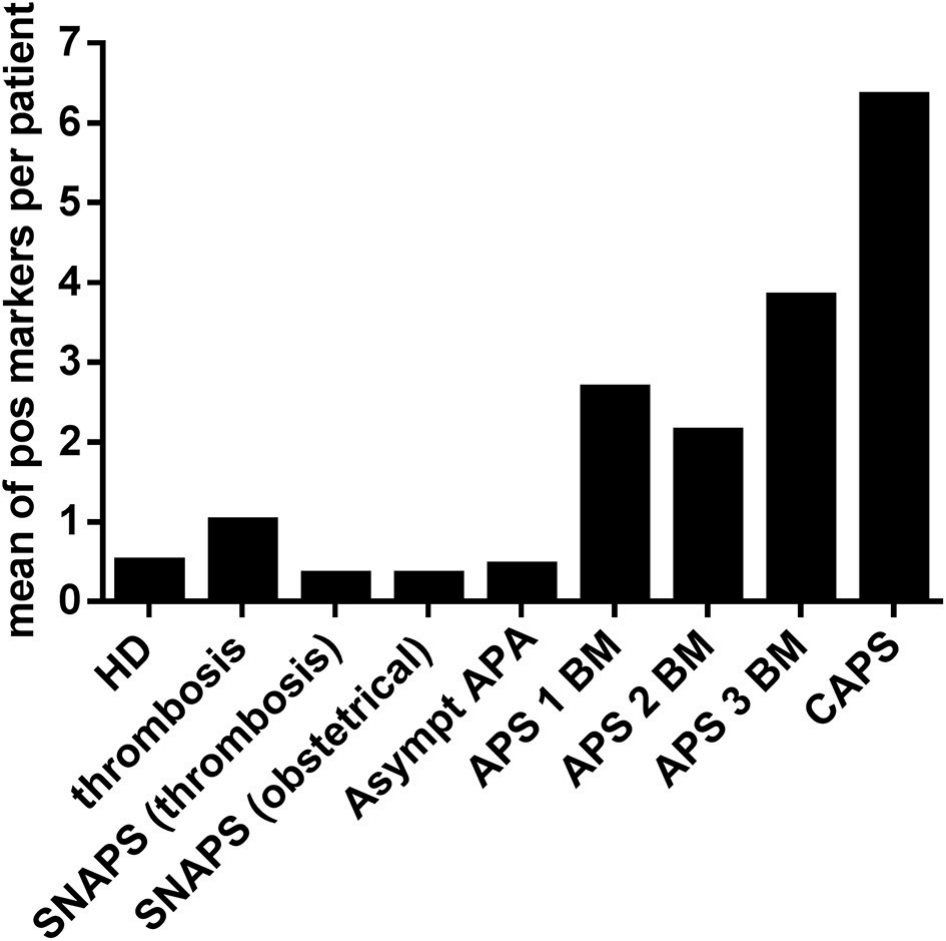
Frequency of other APS non-conventional markers in the different groups of patients and HD. Non-conventional APS markers of IgG and IgM isotypes were detected by immunodot technique in sera of different groups of patients and in HD. Y ax shows a mean of positive markers (PA, PG, PI, PS, A5, and PT of IgG and IgM isotypes) per patient in each group. *HD*, Healthy Donors*; SNAPS*, seronegative anti-phospholipid syndrome*; Asympt APA*, asymptomatic carriers of antiphospholipid antibodies; *APS 1 BM*, APS with 1 biomarker; *APS 2 BM*, APS with 2 biomarkers; *APS 3 BM*, APS with 3 biomarkers; *V thromb*, venous thrombosis; *Art thromb*, arterial thrombosis; *Obst APS*, obstetrical APS; *CAPS*, Catastrophic anti-phospholipid syndrome; *Pos*, positive.

The Figure 3 shows the mean number of positive markers per patient for each group, compared to the HD. In the group of thrombosis, in patients with SNAPS and in the asymptomatic patients with APA, the number of positive markers was inferior or equal to one per patient whereas in APS patients it was from 2 to 6 per patient. The highest number of positive markers was observed in the group of CAPS with a mean of 6.3 IgG or IgM markers per patient when aPS/PT antibodies are excluded, and 10.7 (range from 5 to 14) markers per patient when aPS/PT are included in the analysis. All patients with CAPS were positive for anti-PA and anti-PS IgG antibodies and 2 from 3 patients for anti-PG and anti-PI IgG antibodies. Antibodies of IgM isotype were less frequent in CAPS patients.

### Correlation Between the Different Non-conventional Markers

In APS patients, the highest correlation with conventional markers was observed for IgG directed to PA and PS. We observed a significant correlation between the presence of anti-PA and anti-PS antibodies (Spearman's coefficient of correlation is 0.78 for IgG and 0.75 for IgM), and between anti-PG and anti-PI antibodies (Spearman's coefficient of correlation: 0.84 for IgG and 0.6 for IgM). A significant correlation was also observed between anti-PS, anti-PT, and anti-PS/PT IgM positivity but at lower degree (Spearman test < 0.5, Supplementary Figure 3).

### Clinical Performances of the Tests

The Table 2 summarizes the results of positive and negative predictive values for clinical APS obtained for the aPS/PT, aB2GP1 IgA and aβ2GP1 D1 antibodies. The calculations were performed using a control group composed by the HD and asymptomatic APA carriers.

**Table 2:** Clinical performances of non-conventional APS markers.

	**a/β2GP1 IgA**	**a/β2GP1 domain I**	**a/PS PT IgG**	**a/PS PT IgM**	**PS/PT IgG+IgM**
Sensitivity (%)	38.5 (23.4–55.4)	50 (33.4–66.6)	43.9 (28.5–60.2)	65.8 (49.4–79.9)	43.9 (28.5–60.2)
Specificity (%)	100 (90.7–100)	97.4 (86.5–99.9)	95.1 (83.5–99.4)	73.2 (57.1–85.8)	97.6 (87.1–99.9)
PPV (%)	100	95 (72.8–99.3)	90 (68.8–97.3)	71 (58.6–81)	94.7 (71.6–99.2)
NPV (%)	61.3 (55.3–67)	66.7 (59.2–73.4)	62.9 (56.2–69.2)	68.2 (57.4–77.3)	63.5 (56.9–69.6)
PLR	NA	19.5 (2.74–138.5)	9 (2.23–36.33)	2.45 (1.41–4.26)	18 (2.52–128.6)
NLR	0.62 (0.48–0.79)	0.51 (0.37–0.71)	0.59 (0.45–0.78)	0.47 (0.29–0.74)	0.58 (0.44–0.76)

*Values are shown with 95% CI. For the calculations of predictive values the control group included HD, thrombosis/obstetric and asymptomatic APA groups. PPV, positive predictive value; NPV: negative predictive value; PLR, positive likelihood ratio; NLR, negative likelihood ratio*.

The aPS/PT antibodies of IgM isotype had the highest sensitivity, comparable to the LA sensitivity, while all antibodies of IgG isotype had high specificities. Negative likelihood ratio of aPS/PT antibodies of IgM isotype was the lowest (0.47) while the positive likelihood ratio value was the highest for aβ2GP1 D1 (19.5) and the combination of aPS/PT antibodies of IgG and IgM isotypes (18) (Table 2). The positive likelihood ratio could not be calculated for aβ2GP1 IgA antibodies as their specificity was 100%.

### Non-supervised Hierarchical Clustering of Conventional and Non-conventional APS Markers

**Figure 4:**
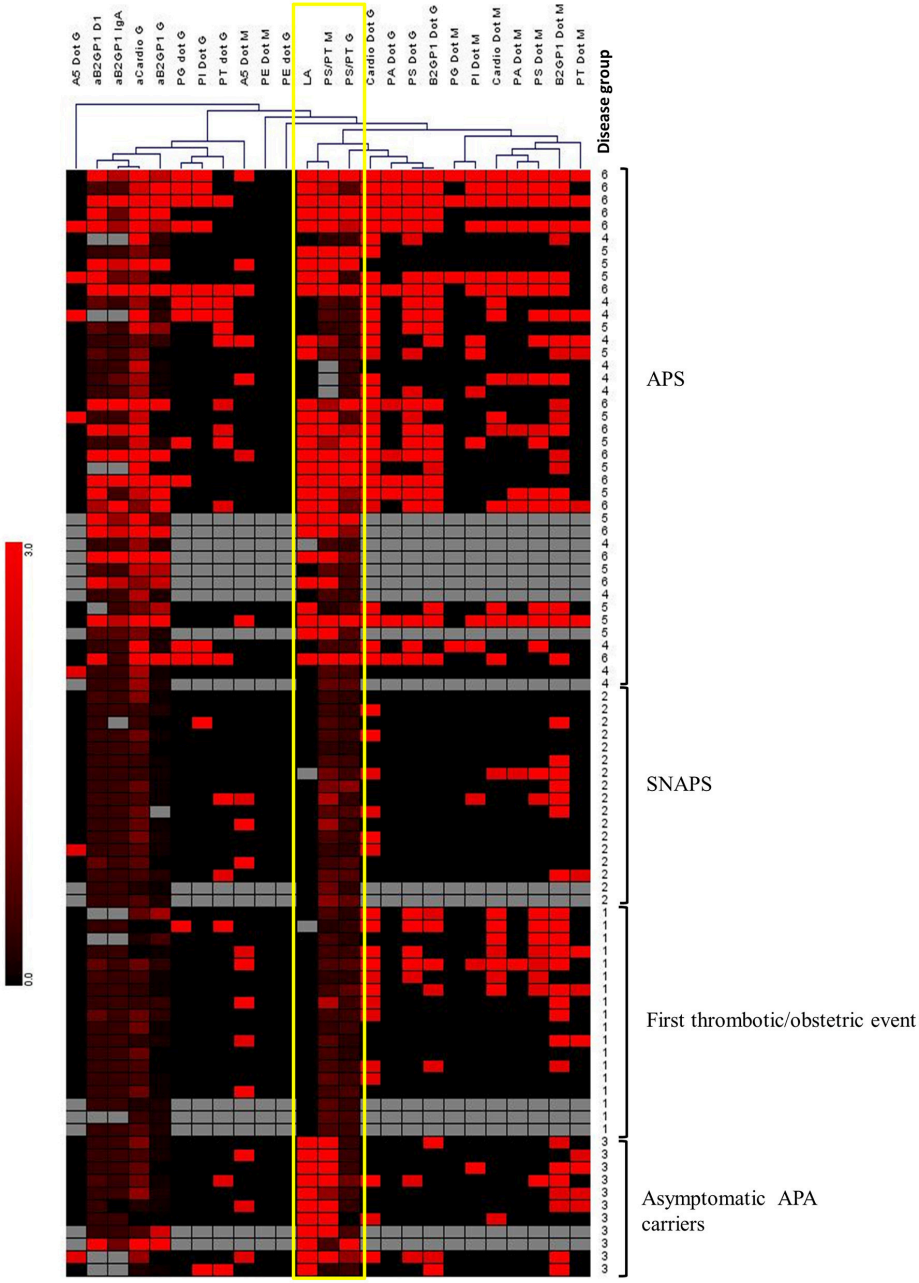
Hierarchical clustering of APS biomarkers using Genesis software. Unsupervised hierarchical clustering was performed, using Pearson correlation and complete linkage analysis. Each column corresponds to an APS marker. Each line corresponds to a patient with a number corresponding to the “disease group”: 1, Thrombosis; 2, SNAPS; 3, asymptomatic APA carrier; 4, APS with 1 biomarker; 5, APS with 2 biomarkers; 6, APS with 3 biomarkers. Colors are correlated with the positivity of the markers from negative (in black) to highly positive (in bright red). Gray boxes correspond to absence of data. This analysis reveals the close clustering of LA with anti-PS/PT IgG and IgM among APS patients, and with anti-PS/PT IgM alone among asymptomatic APA carriers (framed in yellow).

Genesis software v 1.8.1 (30) was used to perform a hierarchical clustering of all tested APS biomarkers in all groups of patients. We used a complete linkage approach and the distances of Pearson correlation. By this method we observed that aPS/PT antibodies of IgM isotype and LA were clustered together (Figure 4). The aPS/PT antibodies of IgG isotype and LA were also clustered in close proximity. In the group of asymptomatic APA carriers only aPS/PT antibodies of IgM isotype and LA were present, whereas in APS group aPS/PT antibodies of IgM isotype and LA were accompanied by aPS/PT antibodies of IgG isotype.

A very strong correlation between the positivity for aPS/PT antibodies of IgM isotype and LA presence was observed (Table 3). Indeed, 35 out of 40 patients with LA (87.5%) were positive for aPS/PT of IgM isotype. When taking into account also IgG aPS/PT, 36 out for 40 patients with LA, were also positive for aPS/PT antibodies (90%).

**Table 3:** Correlation between LA and anti-PS/PT antibodies.

	**IgG anti-PS/PT**	**IgM anti-PS/PT**	**IgG and/or IgM anti-PS/PT**
Sensitivity (%)	50 (33.8–66.2)	87.5 (73.2–95.8)	90 (76.3–97.2)
Specificity (%)	100 (92.1–100)	91.1 (78.8–97.5)	91.1 (78.8–97.5)
PPV (%)	100	89.7 (77.3–95.7)	90 (77.8–95.8)
NPV (%)	69.2 (62.3–75.4)	89.1 (78.2–94.9)	91.1 (80.1–96.3)
PLR	–	9.84 (3.83–25.27)	10.12 (3.95–25.95)
NLR	0.50 (0.37-0.68)	0.14 (0.06–0.31)	0.11 (0.04–0.28)
Chi2	27.4	52.7	55.9

*The performances of anti-PS/PT antibodies were calculated for all patients positive for LA*.

*PPV, positive predictive value; NPV, negative predictive value; PLR, positive likelihood ratio; NLR, negative likelihood ratio; Chi^2^, chi square test*.

### Non-conventional Markers in SNAPS

Among the 17 patients with SNAPS, 9 were positive for at least 1 marker and 4 for more than 1 marker (Supplementary Table 2). Both IgG and IgM isotypes were detected in these patients. The markers found in SNAPS patients were PS/PT, PI, A5, and PT for IgG isotype and PS/PT, PA, PI, PS, A5, and PT for IgM isotype.

IgG aPS/PT were found in 1 patient but they did not persist 12 weeks later. IgM aPS/PT were detected in 3 patients. However, in one of them the antibodies were not persistent; in the two others the persistence was not assessed.

## Discussion

This study aimed to explore the usefulness of several non-conventional APS markers for the diagnosis of APS, to assess whether they might play an additional role for the disease classification or for estimation of disease severity its severity. We have studied the presence and the persistence of IgA aβ2GP1 and IgG aβ2GP1 domain 1 and IgG and IgM directed against the PS/PT complex, and against phosphatidic acid (PA), phosphatidyl-ethanolamine (PE), phosphatidyl-glycerol (PG), phosphatidyl-inositol (PI), phosphatidylserine (PS), annexin V (A5), and prothrombin (PT) in a cohort of patients prospectively constituted. It comprised patients presenting with clinical APS, patients with a first episode of thrombosis and/or APS obstetric criterion but also patients classified as SNAPS and patients with at least one persistent positive APS marker without clinical manifestation.

To date, aPS/PT antibodies are not included in the APS laboratory criteria but their positivity has been recently proposed as a part of the global APS score (GAPSS) (31, 32), and has been shown to be a strong prognostic factor for both arterial and venous thrombosis (33). Our data confirm the high prevalence of aPS/PT antibodies in APS patients (34). We found IgM and IgG aPS/PT in 65.8 and 43.9% of our APS patients, respectively, and their levels were correlated with the number of positive classical markers. All CAPS patients were found positive for IgG and IgM aPS/PT. However, we failed to correlate these antibodies positivity with the type of thrombosis presented by the patients.

Interestingly, there was a striking positivity for IgM aPS/PT in the group of asymptomatic patients with APA (81.8%) whereas a positivity for IgG isotype was not frequent (18.2%). The majority of APA carriers in our study was detected by a pre-surgery assessment of coagulation tests and all were discovered positive firstly for LA. We found a very strong correlation between LA and aPS/PT IgM antibodies (chi-square test = 52.7) and this association was confirmed by our analysis by hierarchical clustering. Previously, it was shown that co-existence of these two parameters is frequent in APS patients (26). Atsumi et al have shown a strong correlation between the presence of aPS/PT antibodies of IgG and IgM isotypes and the presence of LA, whatever the detection method used (34). Our data confirm these findings but also reveal their co-existence in the group of asymptomatic APA carriers in which LA is mainly correlated with presence of IgM aPS/PT. This association might suggest that aPS/PT of IgM isotype may be less pathogenic than those of IgG isotype suggesting that the management of these patients in term of thrombosis prevention and follow up might be less strict.

LA detection method is not accurate for patients treated by DOACs even at low concentration (35). Treatment with DOACs could give false positive results of LA with dilute Russel viper venom time (DRVVT) assay whereas activated partial thromboplastin time (APTT) assay is less influenced (23). It is especially important to be able to replace LA testing, when it could not be measured, with other suitable and similar marker as LA has been shown to be the strongest risk factor of thrombosis comparing to aCL and aβ2GP1 (36). Conventional APA (i.e., LA, aβ2GP, and aCL) triple positivity allows to identify the APS group of patients with persistence of APA (37) and especially with the highest risk of thrombosis or obstetric morbidity recurrence (38, 39). Thus, LA detection is crucial in case of thrombosis' recurrence during the treatment or if suspension of oral anticoagulation is considered (23). Unlike LA detection, anti-PS/PT antibodies are detectable by immunological assay which can be performed on small sample volume of serum or plasma and is not influenced by anti-thrombotic treatments.

So, in these situations, aPS/PT antibodies (IgM and IgG) measuring could be considered to help to confirm or rule out the presence of LA (40).

Despite technical progresses a consistent group of patients with clinical symptoms remains classified as “seronegative.” Recently, even a case of CAPS, has been reported in a patient negative for conventional biological markers (41). This emphasizes the persistent need of other biomarkers of APS. In this objective, we have tested a large panel of other autoantibodies. In contrast to previous studies (21–22, 42), we found aβ2GP1 D1 IgG and aβ2GP1 IgA positivity only in confirmed APS patients with double or triple positivity for conventional markers. These antibodies displayed an excellent specificity but quite low sensitivity and haven't shown any additional benefit as they were not positive in our group of SNAPS patients. One asymptomatic APA carrier exhibited a high positivity for aβ2GP1 D1 antibody. However, this patient had also a triple positivity for conventional APS markers and it was the only patient in this group positive for aPS/PT of IgG isotype. The clinical evolution of this specific patient needs to be carefully monitored.

In our study, all patients positive for aβ2GP1 D1 IgG were positive for aPS/PT antibodies, confirming the correlation between these two markers previously shown by Nakamura et al. in APS patients (43).

The absence of IgA aβ2GP1 positivity in our SNAPS patients contrary to the previous publications (21, 22, 44) may be explained by the small size of our cohort due to the design of our study which was prospective and monocentric, and the quite short period of observation. These antibodies were found only in a small number of studied patients: in about 3% of patients together with conventional antibodies but in < 1% of patients in an isolated manner (44). This may suggest that detection of these antibodies could be helpful in quite limited cases.

We found statistically significant difference in a frequency of non-conventional APS antibodies between patients and HD. These markers were particularly present in APS patients: from 2 to 7 markers per patient (or from 3 to 14 markers per patient if aPS/PT antibodies were taken into account, data not shown), with the highest number of markers in patients with CAPS. As a triple-positive APS characterizes a more severe disease comparing to the double or single positive APS, the positivity for multiple non-conventional markers could be another sign of the disease severity.

We performed a hierarchical clustering of APS markers in patients to study possible association between some markers and patient profiles. As previously suggested, we observed a clusterization of LA and aPS/PT antibodies of IgM isotype in asymptomatic APA group and a clusterization of LA and aPS/PT antibodies of IgG isotype in APS patients. This emphasizes the potential pathogenicity of IgG aPS/PT antibodies. These data are in accordance with recently published study (40) which postulates the pathogenicity of the aPS/PT antibodies of IgG and not of IgM isotype in patients with APS secondary to lupus.

In conclusion, our data obtained from a prospective cohort constituted mainly by patients with primary APS, demonstrate the potential value of aPS/PT antibodies as a strong marker of APS.

## Author Contributions

EL performed the experiments and wrote the manuscript. LD designed the study, included the patients, and wrote the manuscript. AK performed the statistical analysis. YB performed some experiments and reviewed the manuscript. GdL included some patients and reviewed the manuscript. M-AD-D designed the study and wrote the manuscript.

### Conflict of Interest Statement

The authors declare that the research was conducted in the absence of any commercial or financial relationships that could be construed as a potential conflict of interest.
