# Supplementary figures and images for: Prevalence and Significance of Non-conventional Antiphospholipid Antibodies in Patients With Clinical APS Criteria
Source: Front Immunol. 2018 Dec 14;9:2971. doi: 10.3389/fimmu.2018.02971 (PMC6302212; doi:10.3389/fimmu.2018.02971)

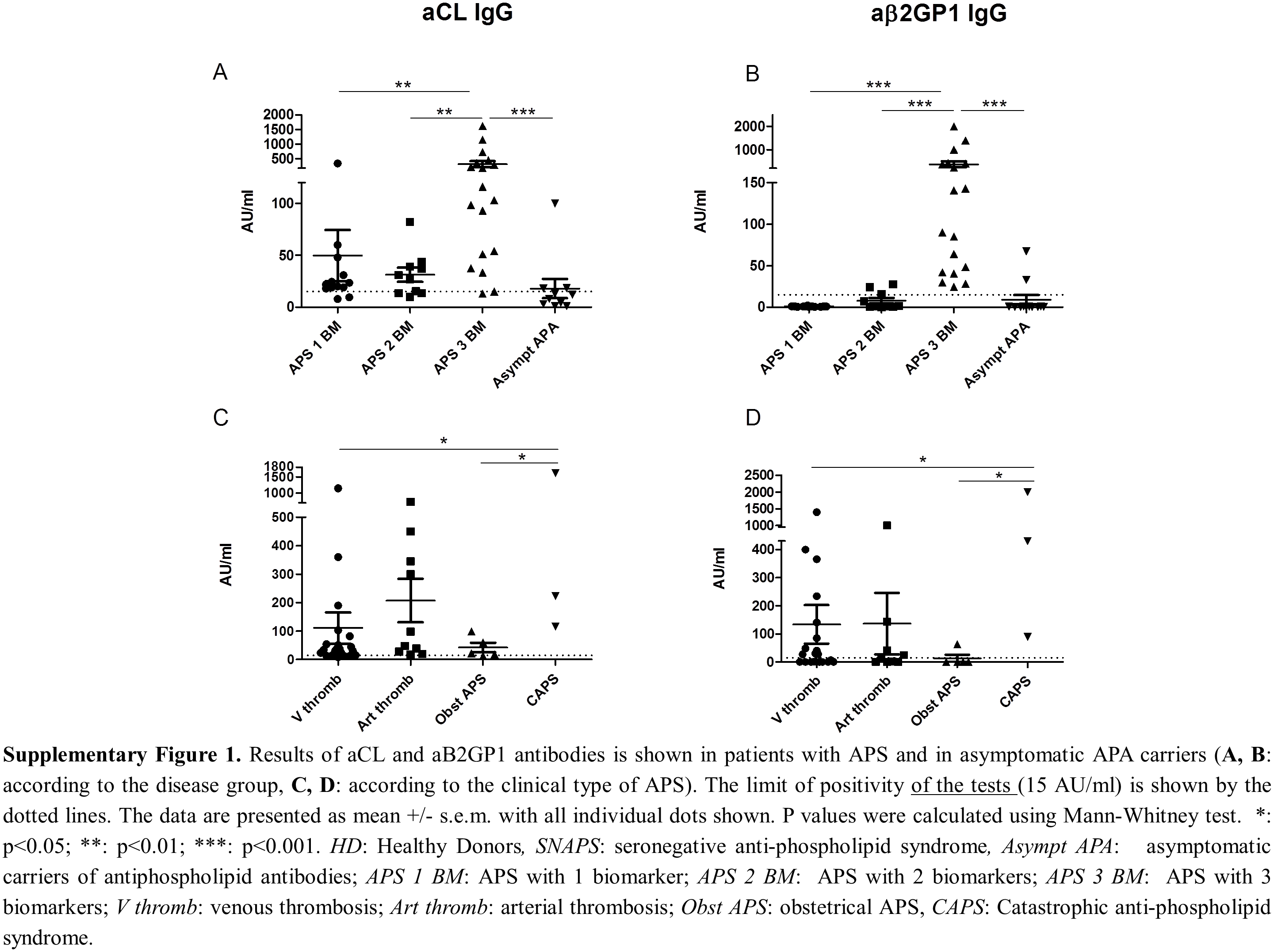

Supplement: Supplementary file 1 [file Image_1.TIF]

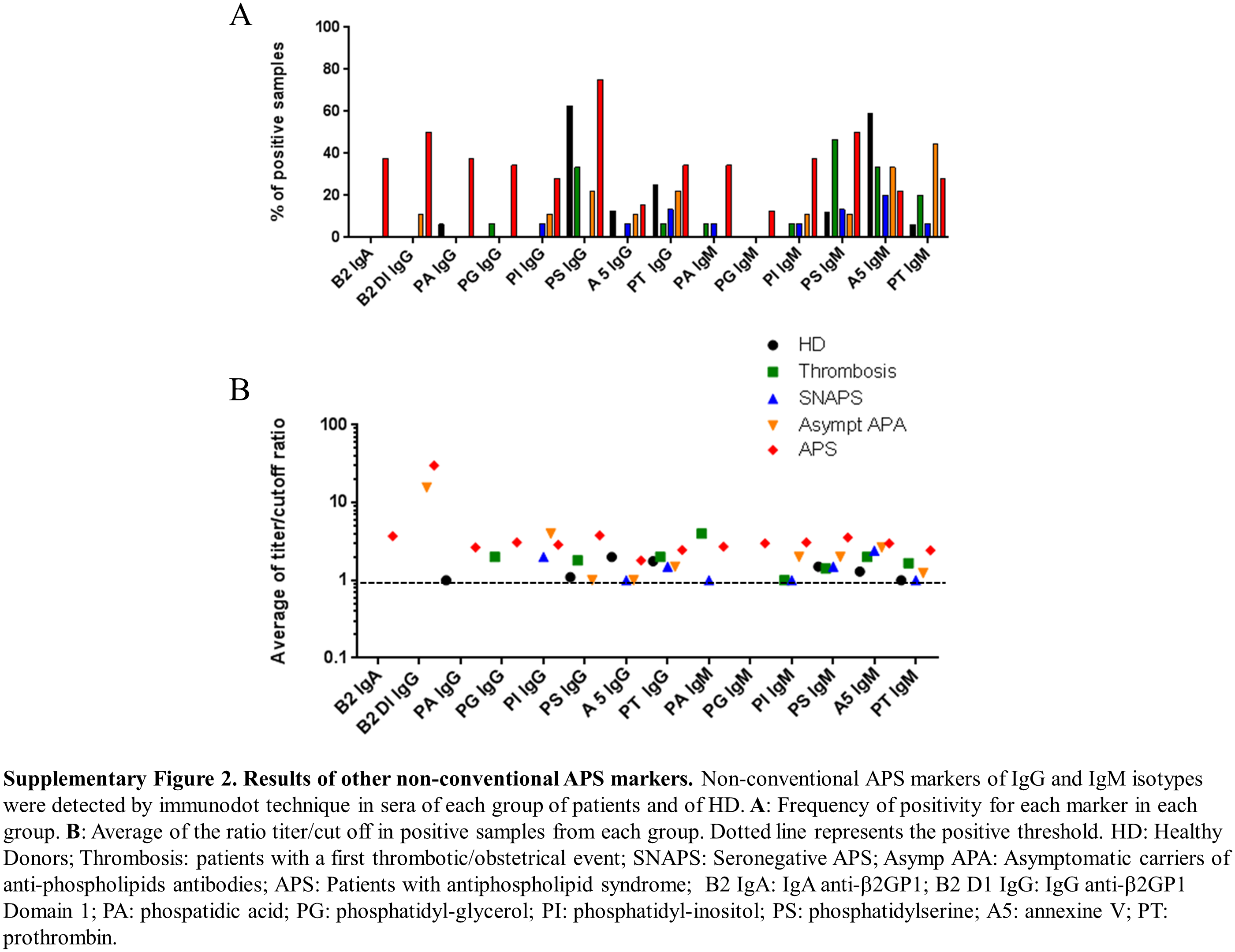

Supplement: Supplementary file 2 [file Image_2.TIF]

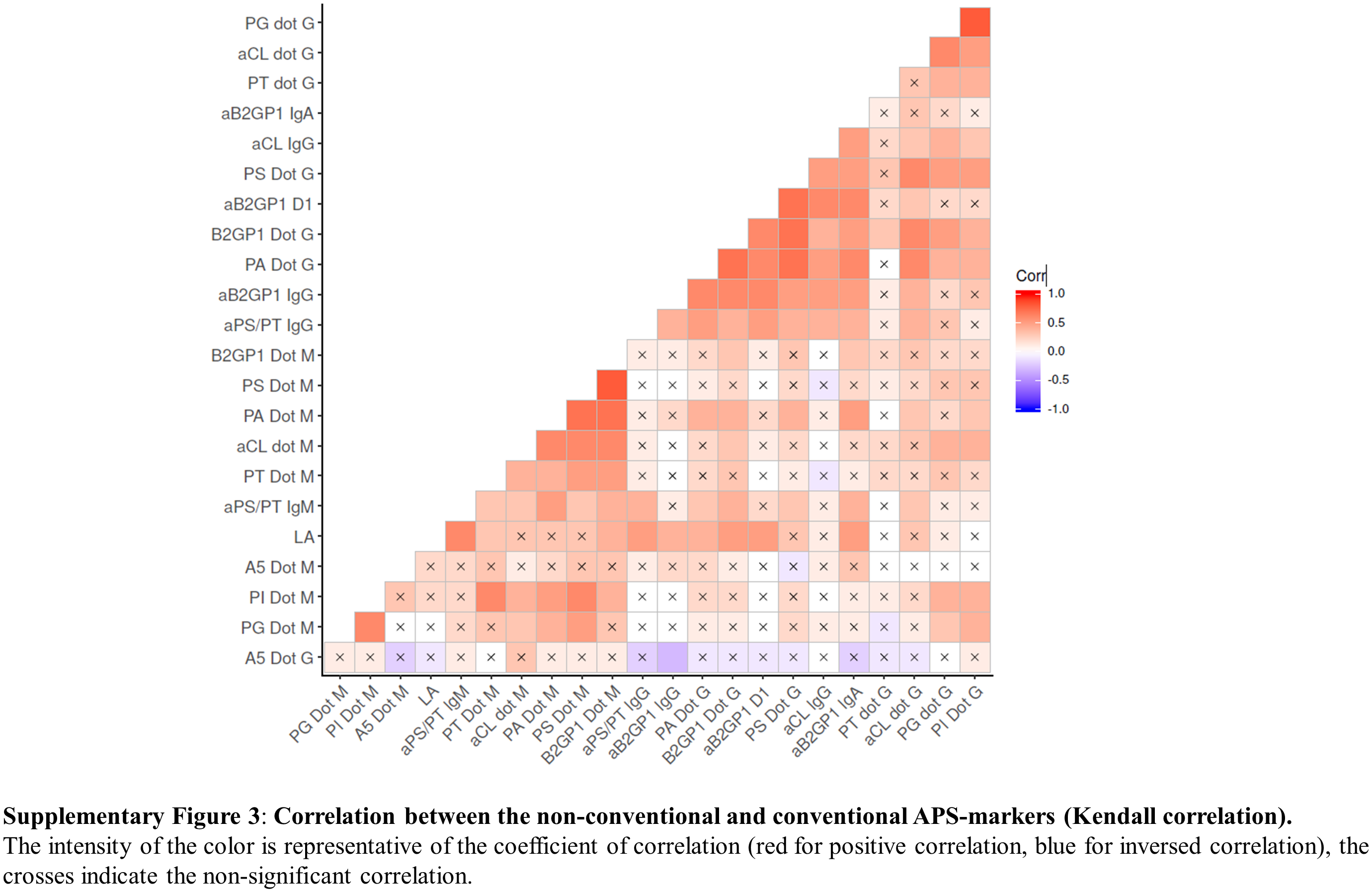

Supplement: Supplementary file 3 [file Image_3.TIF]

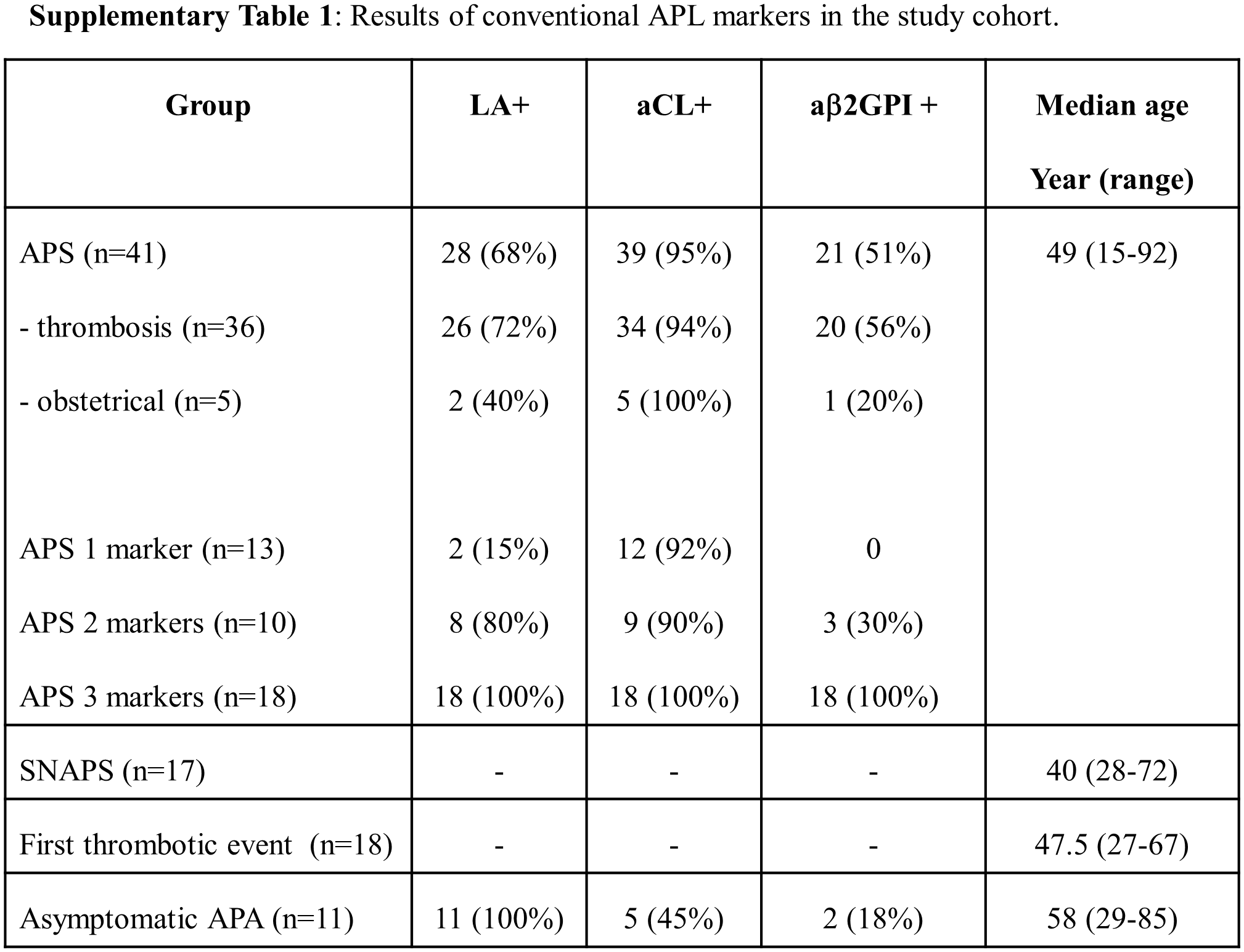

Supplement: Supplementary file 4 [file Image_4.TIF]

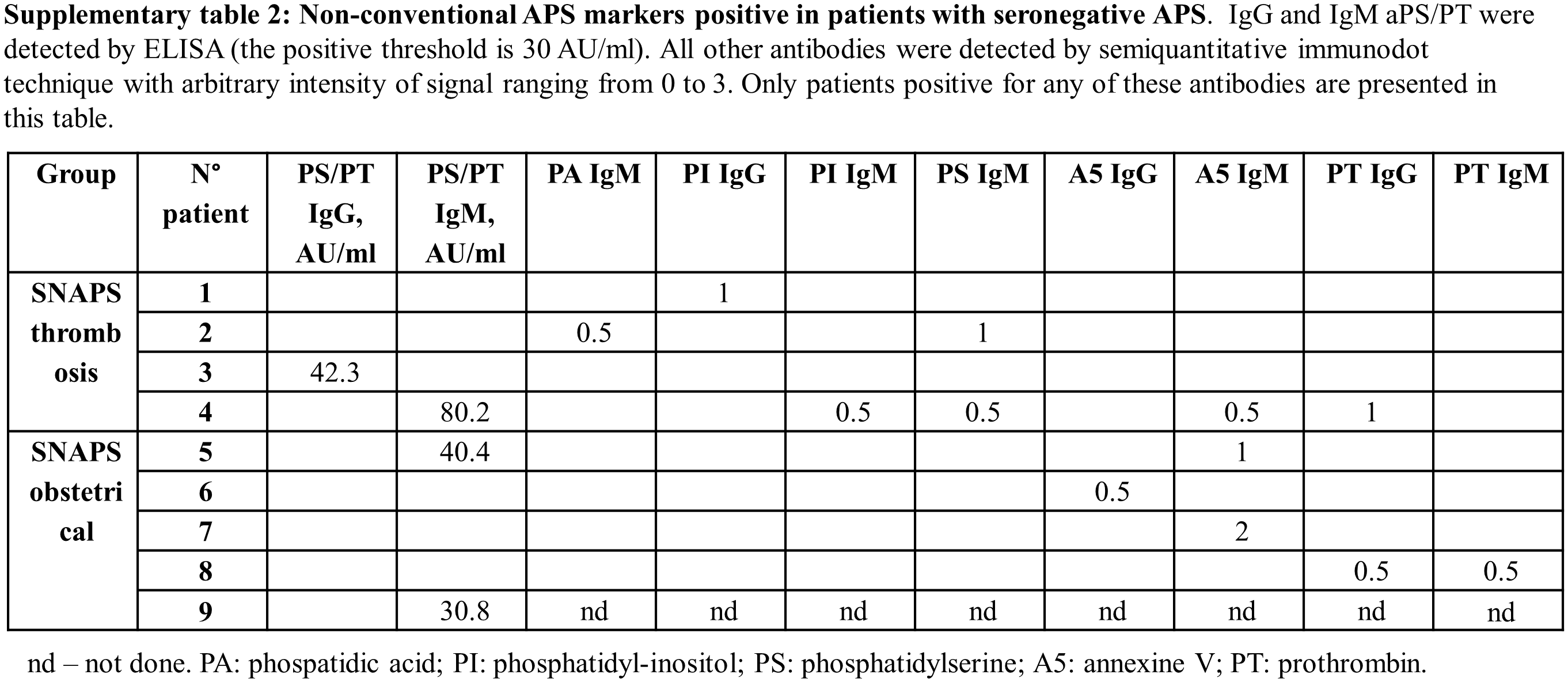

Supplement: Supplementary file 5 [file Image_5.TIF]
